# Fire Prevention and Extinguishing Characteristics of Al^3+^-CS/PAM-MBA Composite Dual-Network Gel

**DOI:** 10.3390/gels11020148

**Published:** 2025-02-19

**Authors:** Jianguo Wang, Yueyang Zhou, Yifan Zhao, Zhenzhen Zhang

**Affiliations:** College of Safety Science and Engineering, Xi’an University of Science and Technology, Xi’an 710054, China; zyy000712@126.com (Y.Z.); 18838536957@163.com (Y.Z.); 15529066075@163.com (Z.Z.)

**Keywords:** coal spontaneous combustion, gel, thermal stability, active groups, fire prevention, extinguishing mechanism

## Abstract

A physically and chemically cross-linked Al^3+^-CS/PAM-MBA dual-network gel with enhanced fire-suppression performance was prepared using chitosan (CS), acrylamide (AM), and N,N’-methylenebisacrylamide (MBA) as base materials. The first network was formed through the covalent cross-linking of polyacrylamide (PAM) with MBA, while the second network was established by crosslinking CS molecules with Al^3+^ ions. The optimal gel ratio was determined by evaluating its formation time and viscosity. The fire prevention and extinguishing performance of the gel was assessed through thermal stability analysis, temperature-programmed studies, infrared spectroscopy, thermal analysis, and fire-extinguishing experiments. The results indicated that the Al^3+^-CS/PAM-MBA dual-network gel exhibited excellent thermal stability and a strong self-ignition inhibition effect, effectively suppressing coal spontaneous combustion and oxidation. The gel achieved this by chemically inactivating coal molecules, disrupting the functional groups closely associated with coal–oxygen reactions and thereby hindering these reactions. Fire-extinguishing tests demonstrated that the gel restrained coal from spontaneous combustion. Upon application, the gel rapidly reduced the coal temperature, making re-ignition less likely.

## 1. Introduction

In recent years, the increasing depth of coal mining and the widespread application of comprehensive mining technologies have significantly increased the risk of coal’s spontaneous combustion in mines [1,2,3,4,5]. This phenomenon not only jeopardizes mine safety, leading to casualties and environmental pollution, but also causes substantial resource waste, undermining the sustainable development of China’s energy system [6,7,8,9,10]. Consequently, the effective prevention and control of coal’s spontaneous combustion has become a critical focus in ensuring coal mine safety in China [11,12].

According to the mechanisms underlying coal’s spontaneous combustion, commonly employed fire prevention techniques include yellow mud grouting [13,14,15], foam [16,17,18,19,20], retardant [21,22], inert gas [23,24,25], and gel [26,27,28,29].

Cao [30] developed a specialized porosity evolution model for high-intensity mining in high-temperature coal seams. Grouting simulation results demonstrated that with flow rates of 300 and 85 m^3^/h, water:cement ratios of 4:1 and 5:1, and drilling spacings of 40 and 48 m, the grouting effect was significant both on the surface and underground. The simulation and optimization results confirmed the effectiveness of these parameters in practical engineering applications.

Zhang [31] developed an innovative three-phase foam fire-extinguishing material by incorporating simulated sea mud particles containing inert materials into the foam. This study not only introduced a new type of three-phase foam with exceptional performance but also outlined a strategy for preparing three-phase foam with high stability and effective fire-extinguishing properties.

Asarapalli [32] investigated the effects of various chemicals, including sodium chloride, calcium carbonate, potassium iodide, sodium nitrate, and potassium chloride, on the spontaneous heating of coal samples. According to the findings, a more effective chemical inhibitor for spontaneous coal heating was proposed. The results from CPT, DTA, and Fourier transform analyses were correlated to identify a superior chemical retardant capable of effectively mitigating the spontaneous combustion tendency of coal.

Liu [33] categorized the coal–oxygen reaction process into five stages based on four characteristic temperatures under N_2_ and CO_2_ conditions. The study found that CO_2_ had a more pronounced effect than N_2_ at the same O_2_ concentration. These findings hold significant value for predicting spontaneous coal combustion and optimizing the use of N_2_ and CO_2_ in coal fire prevention and extinguishing technologies.

Gel fire prevention technology is widely utilized due to its broad applicability, simple and efficient preparation methods, and excellent fire prevention performance [34,35,36,37,38]. Over the past 30 years, countries with rapidly developing mining industries have introduced numerous single-network gel fire prevention materials based on polymer compositions. However, these single-network gels often exhibit poor thermal stability and are prone to plastic deformation or cracking, significantly compromising their fire prevention and extinguishing effectiveness [39,40,41,42,43,44,45]. To address these limitations, Zhou [46] developed a novel biomass gel material using sodium carboxymethyl cellulose (CMC) as the matrix, ferroaluminum citrate (Fe-AlCit) as the crosslinking agent, and glucono-delta-lactone as the pH adjuster. The carboxylate ions in CMC form metal coordination bonds with the high-valent metal ions Fe^3+^ and Al^3+^ dissociated from Fe-AlCit, enhancing the stability of the CMC-based gel through robust metal–ligand interactions. This study offers significant advancements in the development of efficient, environmentally friendly, and self-repairing fire-extinguishing materials for mining applications.

Zhao [47] developed and designed an environmentally friendly, highly stable gel (DF) with a dual cross-linked structure and flame-retardant properties. The gel structure was based on an ionic crosslinking reaction between sodium alginate and Ca^2+^ ions, with sodium carboxymethyl cellulose (CMC) introduced to form additional hydrogen bonds with sodium alginate, creating a physical and chemical dual crosslinking network. Experimental results demonstrated that the DF gel outperformed traditional water-based gels in fire prevention effectiveness.

Yaroslav O. Mezhuev [48] is formed into epoxy-containing copolymers by copolymerization of N-vinyl-2-pyrrolidone and allyl glycidyl ether and is used for dopamine immobilization. The process conforms to the second-order kinetic equation and involves an epoxy group ring-opening reaction. The resulting copolymers form pH-sensitive hydrogels; FeCl_3_ treatment yields pH-responsive gels, while NaIO_4_ oxidation yields complete degradation in pH 7.5 buffer. Artur Krezel [49] studied the coordination behavior of D,L-dithiothreitol (DTT) with Zn(II), Cd(II), Pb(II), Ni(II), and Cu(I). By potentiometric titration, UV-Vis, and NMR spectroscopy, DTT was found to form stable polymer and monomer complexes with these metal ions through its two sulfur donors. This study provides a theoretical basis for predicting the interference of DTT in the study of metal binding of sulfhydryl-containing biomolecules. Anirban Karmakar [50] reviewed the latest advances in urea and thiourea functionalized coordination polymers (CPs) and metal-organic frameworks (MOFs). Due to their conformational flexibility, strong hydrogen bonding capabilities, and polarization characteristics, these materials exhibit excellent performance in gas adsorption and separation, heterogeneous catalysis, and sensing. In this paper, the synthesis methods, structural characteristics, and applications of these functionalized materials are systematically discussed, and the influence of functional groups on the structure and properties of the materials is analyzed.

In the preparation of mine fire prevention and extinguishing gels, although functional groups, such as thiols and catechols, can also form gels with metal ions, the selection of carboxyl and amino polymerization is primarily based on their unique comprehensive advantages: appropriate reaction rates facilitate the control of gelation time, broad pH adaptability and inherent flame retardant properties meet the demands of complex mine environments, while also offering the benefits of readily available raw materials, low cost, safety, and environmental friendliness. These characteristics make the carboxyl/amino system the most suitable chemical choice for mine fire prevention and extinguishing requirements, as it not only meets emergency response needs but also ensures long-term safety and environmental compatibility. Taken together, these factors make carboxyl- and amine-containing polymers the optimal choice for mine fire prevention and extinguishing gels. 

To enhance the water retention and thermal stability of double-network gels for fire prevention, high-density metal ion cross-linkers were incorporated into the system to improve gel inhibition performance. By combining different structured physically crosslinked networks, an Al^3+^-CS/PAM-MBA dual-network gel with outstanding comprehensive properties was developed. Optimal ratios were determined based on gelation time and practical requirements. The fire prevention and extinguishing performance of the gel was evaluated using infrared spectroscopy, programmed heating systems, synchronous thermal analyzers, and custom-designed fire-extinguishing test devices.

## 2. Results and Discussion

### 2.1. Analysis of Gel Base Performance Test

#### 2.1.1. Analysis of Gel Gelatinization

Using gelation time, viscosity, and strength as indicators, a four-factor, three-level orthogonal experiment was conducted with chitosan (CS) content A, acrylamide (AM) content B, N, N’-methylenebisacrylamide (MBA) content C, and a blank control group D as factors. The level codes of each factor are shown in Table 1. The gel formation time test results are shown in Table 2.

**Figure 1:**
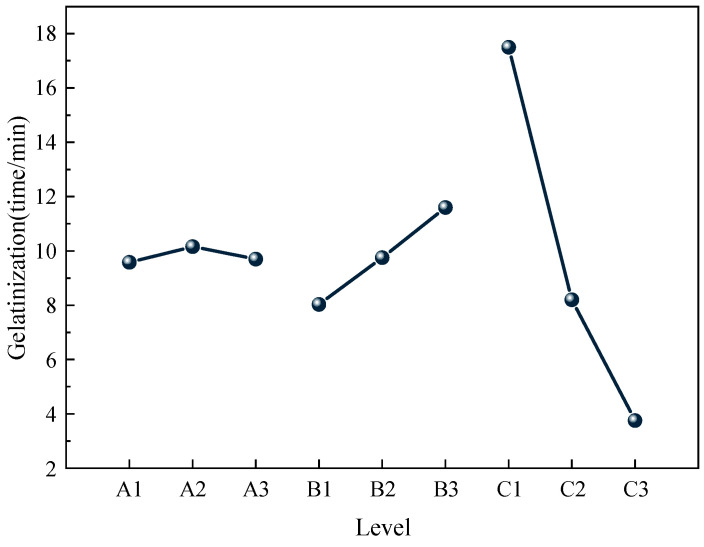
Effect of various factor levels on gelation time.

According to the results of orthogonal experiments, the effects of different factors and levels on the gel forming time were statistically analyzed, and the effects of each factor level on the gel forming time are shown in Figure 1. The results of the significance analysis of each factor are shown in Table 3.

Combined with the chart, it can be seen that the R^2^ value was 0.997, and the closer the R^2^ value was to 1, the more realistic the calculation results. The three factors influencing gel formation time followed the order: MBA > AM > CS. The gel formation time was mainly affected by the MBA. Considering the first network, composed of the PAM molecular chain, as the main network, the gel formation time was mainly influenced by the amount of MBA. Specifically, the higher the content of the crosslinking agent MBA, the faster the gel formation time.

#### 2.1.2. Analysis of Gel Viscosity

The test results are shown in Table 4.

**Figure 2:**
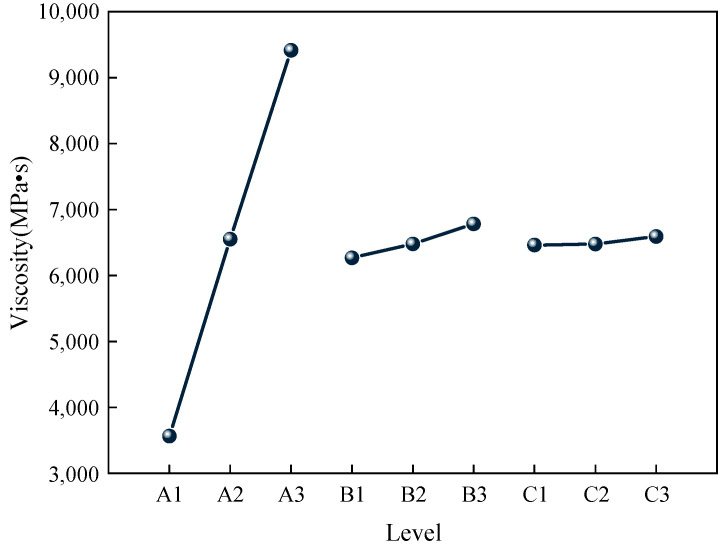
Effect of various factor levels on gel viscosity.

According to the results of orthogonal experiments, the effects of different factors and levels on the viscosity of the gel were statistically analyzed, and the effects of each factor level on the viscosity of the gel are shown in Figure 2. The results of the significance analysis of each factor are shown in Table 5.

The R^2^ value was 0.999, indicating that the calculation results were as expected. Combined with the chart, it can be seen that the effect of the three factors on viscosity followed the order: CS > AM > MBA. Specifically, a higher CS content resulted in a higher viscosity of the gel. This is because CS is a natural polymer with numerous amino and hydroxyl functional groups in its molecular structure, which contribute to a higher viscosity when forming gels in water.

To effectively prevent and control underground fires in coal mines, the gel formation time should neither be too fast nor too slow and should be controlled within the range of 5 to 10 min. Additionally, the gel viscosity should be moderate to ensure both fluidity and permeability. According to the analysis of gel formation time and viscosity, it can be concluded that the gel composed of 2.5 wt% CS, 11 wt% AM, and 0.6‰ MBA dissolved in 2 wt% acetic acid solution demonstrated superior performance. To further improve the crosslinking density and fireproofing ability, a chlorine-based blocking agent, AlCl_3_, was added, resulting in the formulation of a metal ion-enhanced Al^3+^ composite dual-network gel. This gel will be used in the subsequent study of its fireproofing characteristics.

#### 2.1.3. Cross-Linked Network

The first network of the composite double network gel was a chemical network formed by the cross-linking of the covalent bond between PAM and MBA. The second network was achieved by the coordination of amino groups (-NH_2_) and Al^3+^ on the CS molecular chain. The reaction equation was as follows.

(1) nAM+mMBA→Cross-linked PAM network

(2) $$2CS-NH_{2}+Al^{3+}\to CS-NH_{2}\cdot Al^{3+}\cdot NH_{2}-CS$$

### 2.2. Test and Analysis of the Gel Fire-Resistant Properties

#### 2.2.1. Thermal Stability Analysis

**Figure 3:**
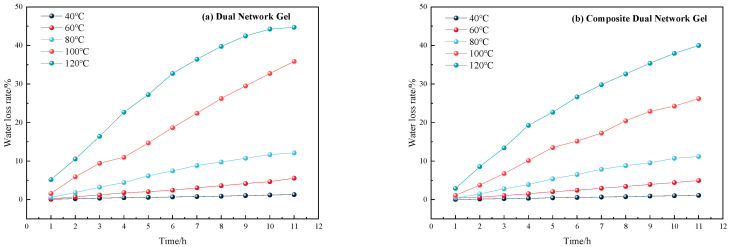
Variation curve of the water loss rate of two gels under a constant temperature.

The water loss rate of the two gels over time at a constant temperature is illustrated in Figure 3.

**Figure 4:**
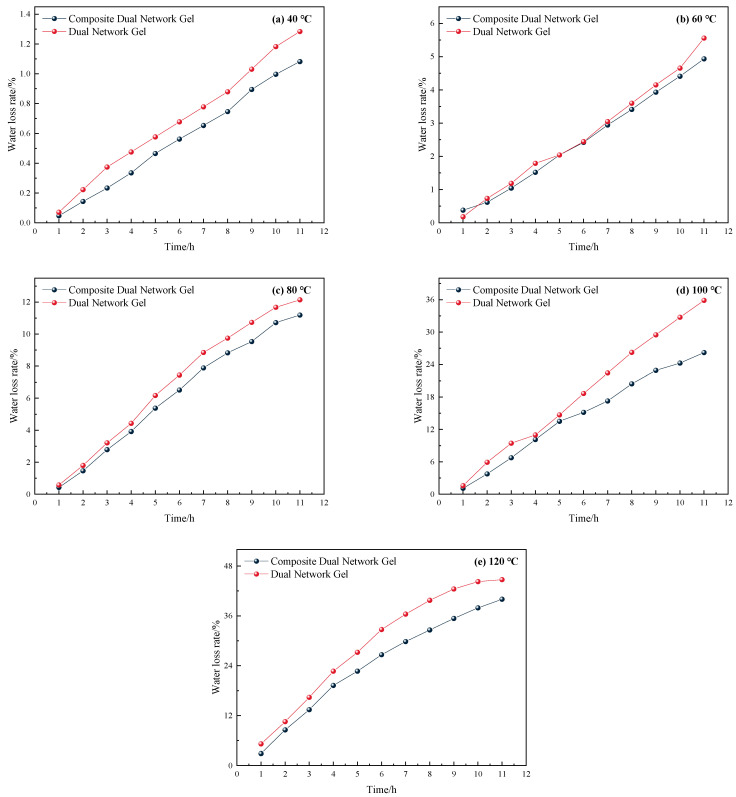
Water loss rate of the two gels under different temperature conditions.

As illustrated in Figure 3, the water loss rate of both gels remained relatively stable and low, below 10%, when the temperature was below 100 °C, with only a slight increase. However, when the temperature reached 100 °C, the water loss rate increased rapidly, reaching approximately 40% after 12 h. The water loss rate changes of the two gels under constant temperature heating at 120 °C are illustrated separately in Figure 4.

As illustrated in Figure 4, under the same temperature conditions, the water loss rate of the composite dual-network gel was significantly lower than that of the dual-network gel. The average water loss rate of the composite dual-network gel was 1% lower than that of the dual-network gel at 40 °C, 4.47% lower at 60 °C, 0.74% lower at 80 °C, 4.23% lower at 100 °C, and 4.83% lower at 120 °C. In conclusion, the composite dual-network gel demonstrated better water retention compared with the dual-network gel.

#### 2.2.2. Analysis of Chemical Resistance Performance Test

(1) Analysis of CO gas change

**Figure 5:**
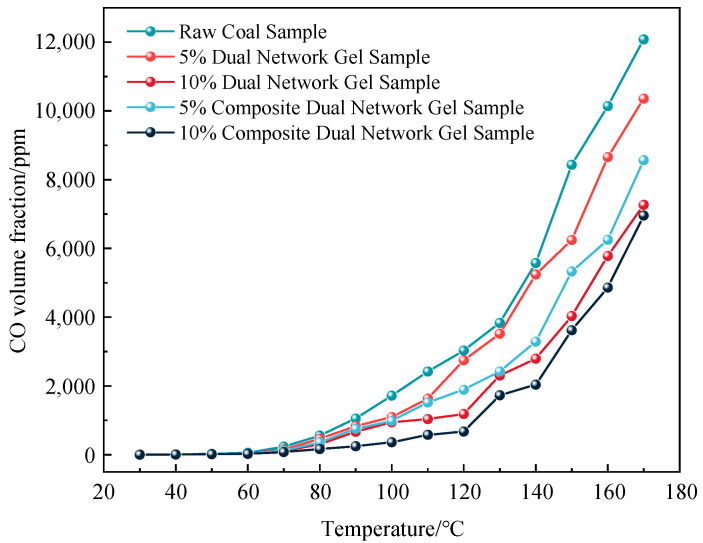
Curve of CO volume fraction as a function of temperature variation.

The changes in CO gas concentration at different temperatures are illustrated in Figure 5.

As illustrated in Figure 5, the CO volume fraction of all five groups of samples increased exponentially. The addition of both gels slowed the low-temperature oxidation of coal and demonstrated a strong inhibitory effect on spontaneous combustion. This inhibition effect was notably enhanced with the increase in gel content. Overall, the composite dual-network gel with the metal ion inhibitor exhibited a significantly better inhibitory effect than the standard dual-network gel without the inhibitor. The observed effects can be attributed to two factors: first, the addition of metal ions increases the crosslinking density of the gel, enhancing its adhesion to the coal and reducing the oxygen supply needed for spontaneous combustion. Second, the high-temperature decomposition of magnesium chloride generates chlorine and other inert gases, which help disrupt or interrupt the combustion chain reaction, thereby inhibiting the combustion process. The incorporation of 5% and 10% metal ion inhibitor composite dual-network gels into the dual-network gel reduced the release of CO by 20.21% and 4.64%, respectively.

(2) Analysis of resistivity

The process of gel inhibition of coal’s spontaneous combustion was qualitatively analyzed by studying the changes in the volume fraction of CO, the signature gas, in different samples during the heating process. However, evaluating the flame-retardant effect of the gel at specific temperature points is challenging. To quantitatively analyze the inhibition of coal spontaneous combustion by gels, the CO retarding rate was calculated by determining the difference in CO volume fraction before and after the low-temperature oxidation process. This calculation was expressed by Equation (3):

(3) $$\eta =\frac{V_{Raw}-V_{Gel}}{V_{Raw}}\times 100\%$$

In the formula, *η* is the blocking rate (%); *V_Raw_* is the CO gas volume fraction of the raw coal (ppm); *V_Gel_* is the CO gas volume fraction of the treated coal samples in each gel experimental group (ppm).

**Figure 6:**
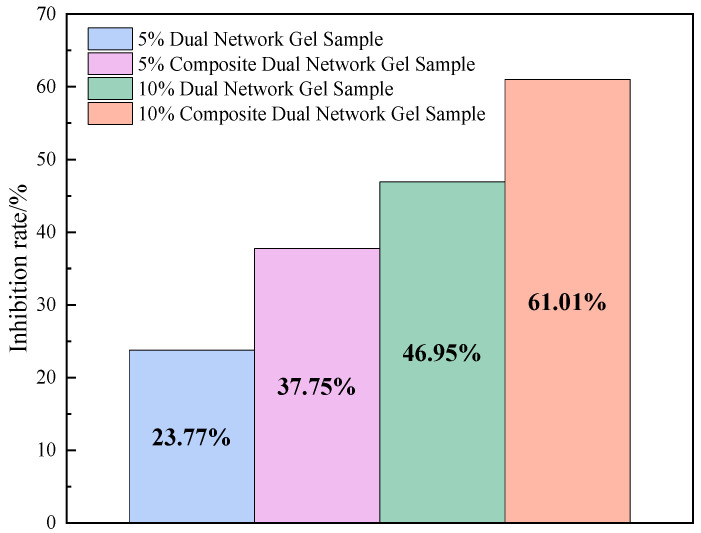
Trend of change in inhibition rate.

The inhibition rates of different samples were calculated using the above equation, as illustrated in Figure 6.

As illustrated in Figure 6, the inhibition effect of the gel on CO was significant, and the composite dual-network gel exhibited a higher inhibition effect than the dual-network gel overall. The average CO inhibition rate for the temperature range of 30–170 °C was calculated as 23.77% for the 5% dual-network gel and 37.75% for the 5% composite dual-network gel, representing an improvement of 14.04%. For the dual-network gel with 10% additive, the CO inhibition rate was 46.95%, while the inhibition rate for the composite dual-network gel with 10% additive was 61.01%, showing an improvement of 14.06%. These results demonstrate that the addition of the Al^3+^ composite dual-network gel significantly enhanced the fire-extinguishing effect, with an approximate increase of 14%.

(3) Activation energy analysis

The size of the activation energy characterizes the difficulty of a reaction occurring. The volume fraction of CO from the coal–oxygen reaction product was analyzed using the reaction rate calculation formula and the Arrhenius equation, and the activation energy of the reaction was determined using the calculation Formula (4):

(4) $$V_{O_{2}}=\frac{V_{CO}}{m}=Ac_{O_{2}}^{n}exp(\frac{E}{RT_{i}})$$

In the formula, m is the CO stoichiometric number; A is the finger front factor; $c_{O_{2}}$ is the oxygen integral number (ppm); n is the number of reaction steps; E is the apparent activation energy of the coal sample reaction, J/mol; R is the universal gas constant, taken as 8.314 J/(mol·K); and *T_i_* is the thermodynamic temperature of coal.

The collation was obtained as Equation (5):

(5) $$lnc_{t}=\frac{E}{RT_{i}}+ln(\frac{mSALc_{O_{2}}^{n}}{kV_{g}})$$

**Figure 7:**
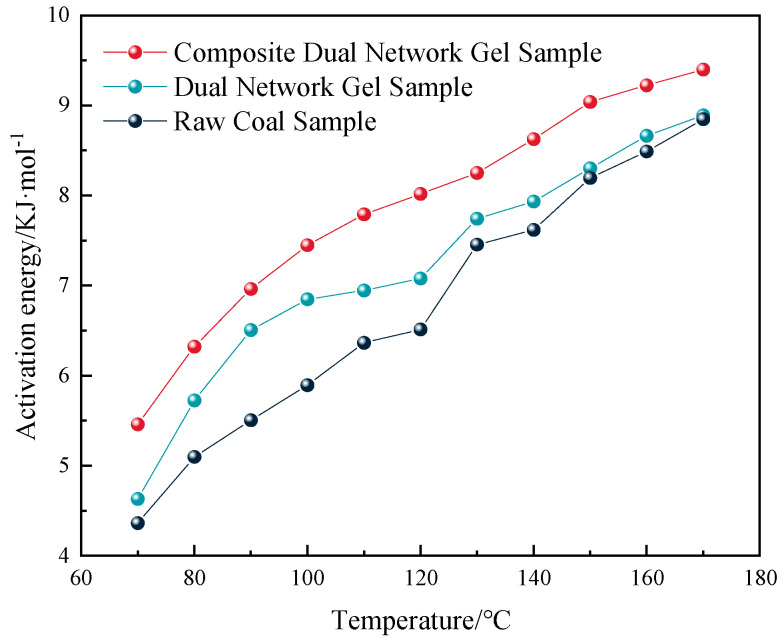
Curve of activation energy for each group with temperature variation.

In the formula, k is the unit conversion factor, taken as 22.4 × 10^9^; *V_g_* is the gas flow rate, m/s; $c_{t}$ is the CO volume fraction for each group of coal samples in ppm. By calculating the slope using this formula, the apparent activation energy of coal samples at different reaction stages was determined, and the calculation results are illustrated in Figure 7.

As illustrated in Figure 7, throughout the warming process, compared with the original coal group, the activation energy of the oxidation reaction for coal samples treated with different gels showed varying degrees of enhancement, with increases of 9.8% and 23.4%, respectively. This indicates that the gel material increased the activation energy required for the coal spontaneous combustion reaction, making it more difficult for such a reaction to occur. The composite dual-network gel exhibited a better inhibition effect compared with the dual-network gel.

#### 2.2.3. Infrared Spectroscopy Analysis

**Figure 8:**
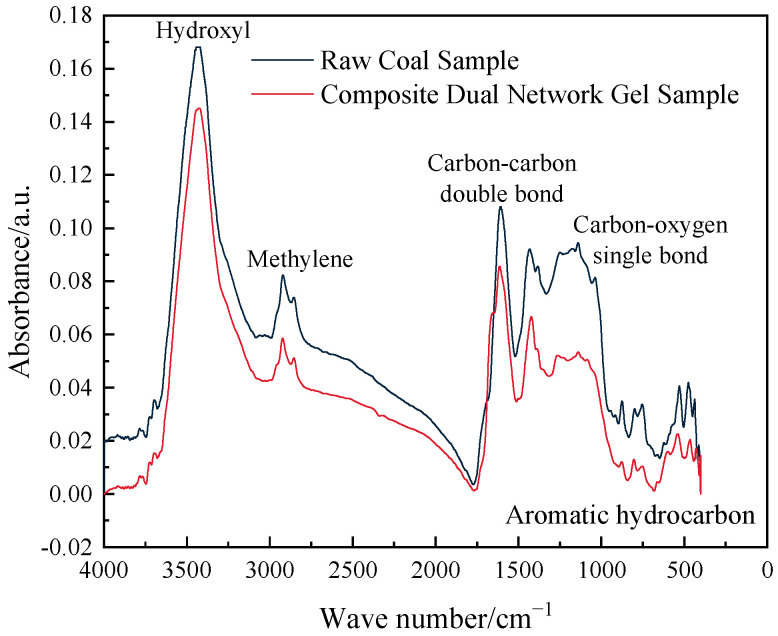
Infrared absorption spectra of different sample groups.

The infrared absorption spectra of the two groups of samples are illustrated in Figure 8.

As illustrated in Figure 8, the infrared absorption spectra of the two groups of samples exhibited similar morphology, with slight differences in peak heights and positions. The infrared spectroscopy results indicate that the composite dual-network gel inhibited aromatic hydrocarbons, aliphatic hydrocarbons, and oxygen-containing functional groups in raw coal to varying degrees. The addition of Al^3+^ in the composite dual-network gel effectively induced electronic shifts in the functional groups, leading to the breakage of covalent bonds. By disrupting the functional groups closely involved in the coal–oxygen reaction, the coal molecules become chemically inert, making it more difficult for the coal–oxygen reaction to proceed, thereby enhancing the inhibition of coal spontaneous combustion.

#### 2.2.4. Characterization of Mass and Heat Changes

**Figure 9:**
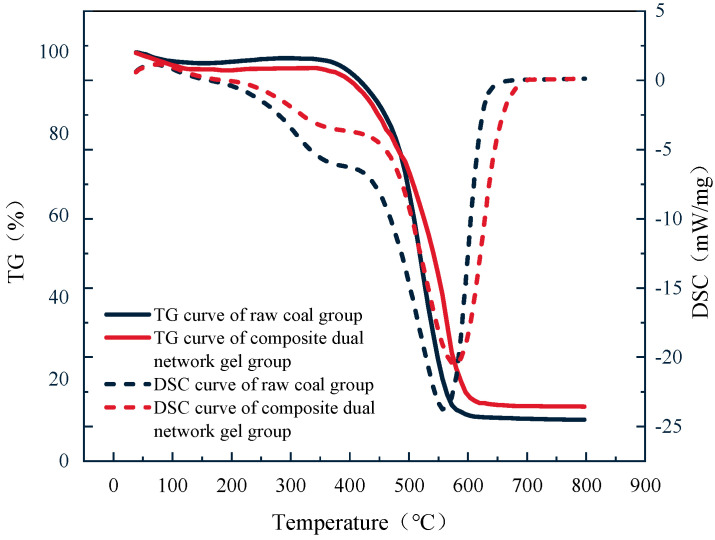
TG-DSC curve of raw coal and composite dual-network gel-treated coal samples.

The TG–DSC curves for the original coal and the treated coal samples are illustrated in Figure 9.

As illustrated in Figure 9, the TG–DSC curves of the original coal and the gel-treated group were generally similar, though some differences were noted. During the water evaporation and desorption stage, the weight loss of the gel-treated group was faster, likely owing to the rapid loss of water in the gel. In the oxygen uptake and weight gain stage, the gel acts as an oxygen barrier, resulting in less weight gain. During the thermal decomposition stage, the internal structure of the gel was damaged, leading to a more significant weight loss. In the later stage of combustion, the changes in the gel-treated group and the original coal were essentially the same.

The DSC curves of the gel-treated group lagged behind the original coal in the first four stages. This indicates that the gel exhibited strong water-holding and water-retaining properties during this process, which helped inhibit the coal–oxygen reaction.

#### 2.2.5. Analysis of Fire-Extinguishing Test

**Figure 10:**
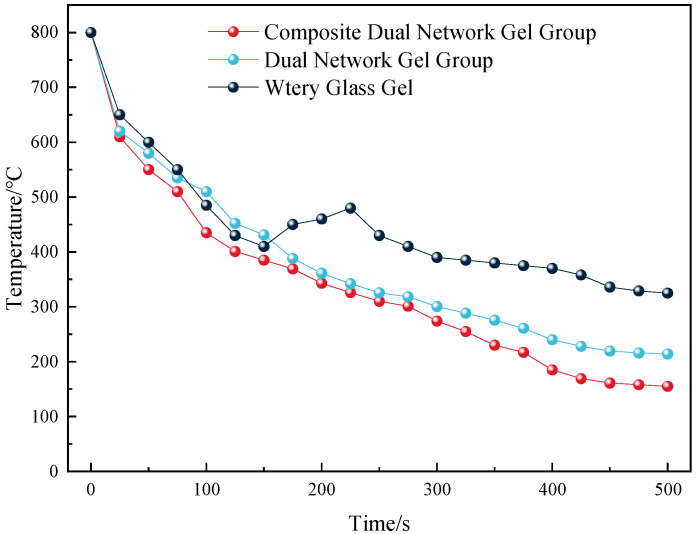
Comparison of fire-extinguishing effects of different gel treatments.

The fire-extinguishing effects of the composite dual-network gel with metal ion inhibitor, the dual-network gel without metal ion inhibitor, and the water glass gel are illustrated in Figure 10.

As illustrated in Figure 10, all three types of gels significantly reduced the coal body temperature. However, the water glass gel exhibited a brief temperature rise during the fire prevention process caused by the rekindling of the coal body. This phenomenon is attributed to the water loss and cracking of the water glass gel under high temperatures, which reduces its ability to cover the coal body and isolate oxygen, leading to air leakage and spontaneous combustion. In contrast, the composite dual-network gel demonstrated a notably better cooling effect than both the water glass gel and dual-network gel, reducing the coal body temperature to below 200 °C within 500 s.

### 2.3. Flame Retardant Mechanism Analysis

The flame retardant mechanism of the composite dual-network gel mainly involves three aspects: water retention and hydration, oxygen isolation, and the reduction of coal susceptibility to spontaneous combustion.

(1) Water locking and moisturizing

The dual-network gel is composed of 80% water, which helps moisturize the coal seam. However, owing to the high permeability of water molecules, it cannot provide continuous temperature reduction on its own. The unique dual-network and pore structure of the gel enhances its water absorption capacity, effectively locking in moisture. This action helps cool the coal during spontaneous combustion, extending the heat absorption and cooling process until the coal is fully extinguished.

(2) Oxygen isolation

A portion of the gel remains in the cracks, effectively isolating oxygen, while another portion penetrates the fire area and comes into contact with the coal. The dual-network structure enhances the gel’s resistance to destruction and increases its compressive strength, making it less likely to be destroyed after bonding with the coal. This continuous oxygen isolation slows down the coal–oxygen reaction, reducing the likelihood of spontaneous combustion and helping to suppress the ignition of coal.

(3) Reduction of coal’s tendency to spontaneously combust

The dual-network gel increases the activation energy of the coal–oxygen reaction, thereby raising the ignition point temperature and reducing CO gas generation. The Al^3+^ ions in the gel reduce the activity of coal molecular groups, improving their chemical inertness. They also inhibit aromatic hydrocarbons, aliphatic hydrocarbons, and oxygen-containing functional groups, especially those closely related to the coal’s spontaneous combustion. By disrupting these functional groups, the gel makes coal molecules chemically inert, thus decreasing their propensity for spontaneous combustion.

**Figure 11:**
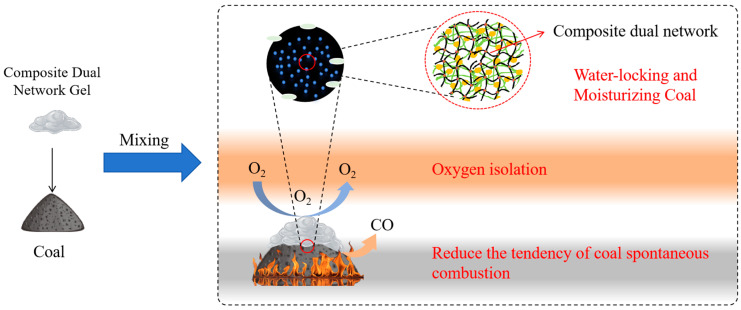
Diagram of the flame retardant mechanism of the composite dual-network gel.

The flame retardant mechanism of the composite dual-network gel is illustrated in Figure 11.

## 3. Conclusions

(1) Through the variation of the concentrations of CS, PAM, and MBA and measurements of gel formation time, viscosity, and other properties, the optimal composition was determined to be 2.5 wt% CS, 11 wt% PAM, and 0.6‰ MBA dissolved in a 2 wt% acetic acid solution. A chlorine-based resistive agent, AlCl_3_, was then added to prepare a metal ion-enhanced Al^3+^ composite dual-network gel.

(2) Thermal stability analysis revealed that the composite dual-network gel had a 10% lower water loss rate compared with the dual-network gel, indicating better water-locking properties. Inhibition performance analysis showed that CO release was reduced after treatment with the gel, and the composite dual-network gel with the metal ion inhibitor exhibited better inhibition than the dual-network gel without the inhibitor. Activation energy calculations showed that the activation energy of the gel-treated coal samples was higher than that of the original coal group, suggesting that the gel increased the activation energy of the coal–oxygen reaction, making spontaneous combustion more difficult. The composite dual-network gel demonstrated superior performance in this regard.

(3) Infrared spectroscopy analysis revealed that the addition of Al^3+^ in the composite dual-network gel effectively induced electronic shifts in the functional groups, leading to the breakage of covalent bonds. This process destroyed the functional groups closely associated with the coal–oxygen reaction, making the coal molecules chemically inert and more resistant to the coal–oxygen reaction, thereby inhibiting spontaneous combustion. TG–DSC analysis showed that the composite dual-network gel exhibited strong water retention and water solidification compared with the original coal. Fire extinguishing tests demonstrated that the addition of the composite dual-network gel significantly reduced the fire source temperature and prevented re-ignition.

(4) The flame retardant mechanism of the composite dual-network gel mainly involved three key aspects: water locking and moisturizing, oxygen isolation, and reduction of the coal’s tendency for spontaneous combustion.

## 4. Materials and Methods

### 4.1. Experimental Materials and Instruments

The experimental materials included chitosan (CS) with a deacetylation degree of ≥95%, N,N’-methylenebisacrylamide (MBA), analytically pure; acrylamide (AM), analytically pure; polyaluminum chloride (PAC), industrial grade; glacial acetic acid (CH_3_COOH), citric acid (CA), potassium persulfate (KPS), and tetramethylethylenediamine (TEMED), all analytically pure.

### 4.2. Preparation Steps

**Figure 12:**
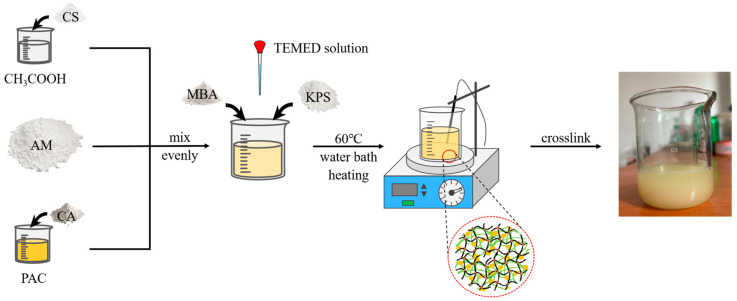
Preparation process for Al^3+^-CS/PAM-MBA composite dual-network gel.

First, 2.5 wt% CS was dissolved in a 2 wt% acetic acid (CH_3_COOH) solution and stirred thoroughly for later use. Afterward, CA was slowly added to a polyaluminum chloride solution to prepare an aluminum citrate (AlCit) solution, which was then stirred quickly for later use. The two prepared solutions were mixed with 11 wt% AM, stirred uniformly, and then methylenebisacrylamide (MBA), KPS, and a small amount of TEMED solution were added to initiate the polymerization reaction. The resulting solution was allowed to stand for 30 min to remove any bubbles generated during stirring and then placed in a 60 °C water bath to accelerate the polymerization process, resulting in the Al^3+^-CS/PAM-MBA dual-network gel. The preparation process is illustrated in Figure 12.

### 4.3. Basic Performance Testing of Gel

#### 4.3.1. Gelation Time

Gelation time is a critical indicator of the gel’s fire prevention performance. If the gelation time is too rapid, the gel will not penetrate deeply into the coal seam. Conversely, if the gelation time is too slow, the gel will not remain in the cracks, reducing its oxygen isolation effectiveness. Nine different concentration ratios of the gel were designed through orthogonal experiments, and gelation time was measured using the drop time method. The test error of gelatinization time was within the range of 0.1 min.

#### 4.3.2. Gel Viscosity

Viscosity is a key indicator of the gel’s ability to remain in coal fractures. To measure the viscosity before and after gelation, a No. 4 rotor was used at a speed of 60 r/min to test each gel sample. To improve the accuracy of the experiment, viscosity values were recorded every minute until no significant fluctuations were observed. The viscosity test error was in the range of 5 MPa·s.

### 4.4. Gel Fire Prevention and Extinguishing Performance Testing

#### 4.4.1. Thermal Stability Testing

The thermal stability of the gel plays a crucial role in the prevention and control of mine fires. Spontaneous combustion of coal generates high-temperature zones, and if the gel is applied directly to these areas, its poor stability can cause rapid drying, cracking, and thermal decomposition, significantly reducing its fireproofing effectiveness. Therefore, studying the thermal stability of the gel is an essential factor in determining whether the gel fireproof material can effectively prevent coal’s spontaneous combustion.

The thermal stability of the gel is usually reflected by the water retention rate because 80% of the material that makes up the gel is water, and the water will evaporate in a continuous high temperature environment. Thus, the temperature is the main factor affecting the thermal stability of the gel. In the process of coal mine fire prevention, the gel adsorbs a large amount of water to cover the surface of the coal body and carries out continuous wetting and cooling effects on the coal seam. If the thermal stability of the gel is poor, in the process of increasing the temperature of the coal body, a large amount of water will be lost, which will greatly reduce the fire prevention and extinguishing effects of the gel. Therefore, it is necessary to determine the water retention capacity of the gel. The water retention rate of the gel was generally expressed by subtracting the water loss rate from unit 1, and the water loss rate of the gel was measured and tested by a constant temperature drying oven.

Using a blast drying oven, the temperature was set to 40 °C, 60 °C, 80 °C, and 100 °C. A 100 g sample of each of the two preferred gels was placed in the oven for 12 h. The gel mass was measured every hour during the test, and the water loss rate of the gels was calculated using Formula (6). The water loss rate was then used to characterize the thermal stability of the gels.

(6) $$W_{L}=\frac{m_{0}-m_{i}}{m_{0}}\times 100\%$$

In the formula, *W_L_* is the water loss rate (%); *m_0_* is the initial gel mass (g); *m_i_* is the water loss gel mass (g). The water loss rate test error was within 1%.

#### 4.4.2. Resistance Performance Test

**Figure 13:**
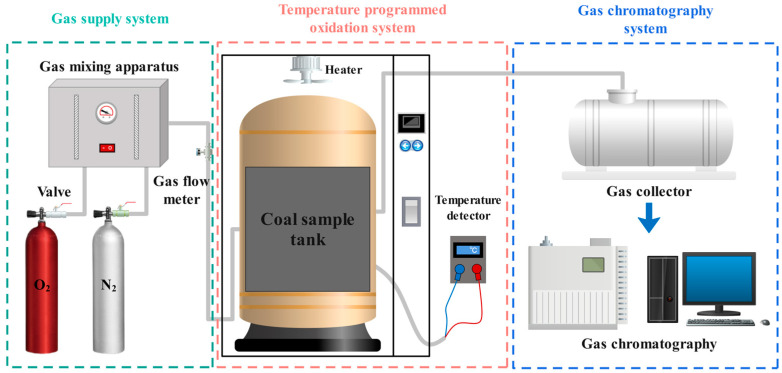
Diagram of the programmed heating device.

The test was conducted using the programmed heating and oxidation system illustrated in Figure 13. Raw coal and gel-treated coal samples were selected for comparative analysis of inhibition performance. A mixed particle size of 200 g from each of the 5 coal samples (0–0.9 mm, 0.9–3 mm, 3–5 mm, 5–7 mm, and 7–10 mm) was vacuum dried at 35 °C for 12 h. The gas tightness of the gas circuit was checked, and the airflow rate through the coal sample tank was set to 120 mL/min. The temperature range was set from 30 °C to 170 °C, with a heating rate of 1 °C/min. Gas samples were collected every time the temperature increased by 10 °C. These gas samples were then injected into a chromatograph to measure the changes in the CO gas concentration at different temperatures. The error of the programmed heating experiment was within the range of 3 ppm.

#### 4.4.3. Infrared Spectroscopy

The coal samples treated with water and the composite dual-network gel were dried at 30 °C for 48 h, while potassium bromide was dried at 105 °C for 3 h. The samples were then pressed. The experimental test wave number range was 4000–400 cm^−1^, and the sample scanning frequency was set to 32 scans per second. The infrared spectra of the two groups of samples were then tested. The error of infrared spectroscopy was in the range of 1 absorbance.

#### 4.4.4. Mass and Heat Change Characterization Test

A thermogravimetric analysis–differential scanning calorimetry (TG–DSC) Japan HITACHI STA200 synchronized thermal analyzer was used for the test. Raw coal and composite dual-network gel-treated coal samples were selected for thermogravimetric analysis. Nitrogen was introduced into the heating furnace at a flow rate of 50 mL/min. The experimental temperature range was set from 35 °C to 800 °C, with a heating rate of 5 °C/min. The test was conducted in a nitrogen atmosphere, maintaining a nitrogen flow rate of 50 mL/min, with the temperature range set from 30 °C to 800 °C and a heating rate of 5 K/min. The TG test error was within 1%, and the DSC test error was within the range of 0.5 mW/mg.

#### 4.4.5. Fire-Extinguishing Test

**Figure 14:**
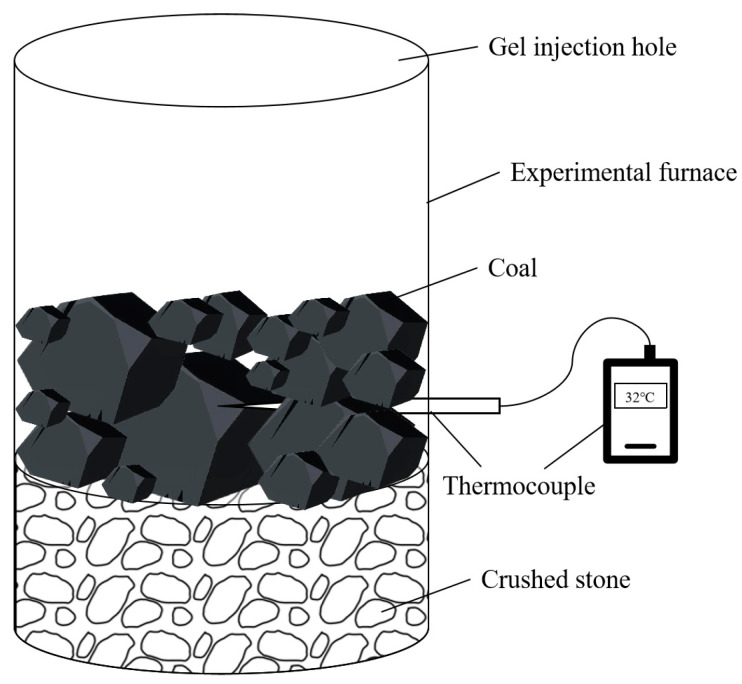
Homemade fire-extinguishing test device.

The actual fire-extinguishing effect of the gel was tested using a homemade test device. A coal block was ignited to simulate coal‘s spontaneous combustion. When the coal temperature reached 800 °C, water or gel was injected into the device, and the temperature was recorded every 10 min until it stabilized. The fire-extinguishing effects of water, the double-network gel, and the Al^3+^ composite double-network gel were then compared. The test device is illustrated in Figure 14. The error of the fire extinguishing test was within the range of 3 °C.

### 4.5. Economic Analysis

The unit prices of the materials used in the composite double network gel are shown in Table 6.

As can be seen from Table 1, the materials with higher unit prices were CS, MBA, and TEMED, among which MBA and TEMED only played the role of initiating the polymerization reaction in the preparation of gels, and the amount required was very small. Although CS was the main component of the gel, the amount used in the preparation process was still not high, and the content was only 5 wt%. When used at the same time, the composite double-network gel had excellent flame retardant properties and did not produce any harmful by-products or residues that could have affected the environment or human health during preparation and use. In the future, we will continue to develop low-cost preparation processes and explore recycling options for CS.

## Figures and Tables

**Table 1:** Orthogonal test factor level table.

Level	Factor			
	A	B	C	D
	CS Content/%	AM Content/%	MBA Content/‰	Blank Group
1	2	10	0.4	1
2	2.5	11	0.6	2
3	3	12	0.8	3

**Table 2:** Orthogonal experimental design and gelation time.

Number of Experiment	CS Content (%)	AM Content (%)	MBA Content (%)	Gelatinization (Time/min)
1	2	10	0.4	15.1
2	2	11	0.8	3.8
3	2	12	0.6	10.1
4	2.5	10	0.8	2.5
5	2.5	11	0.6	8.2
6	2.5	12	0.4	20.1
7	3	10	0.6	6.6
8	3	11	0.4	17.5
9	3	12	0.8	5.3

**Table 3:** Significance analysis of CS, AM, and MBA content on gelation time.

Source of Differences	Sum of Squares of Dispersion	Degree of Freedom	Mean Square	F	*p*
CS	0.572	2	0.286	0.533	0.652
AM	19.822	2	9.911	18.496	0.051
MBA	295.355	2	147.677	275.603	0.004

R^2^ = 0.997, *p* < 0.05 Significant.

**Table 4:** Orthogonal experimental design and viscosity.

Number of Experiment	CS Content (%)	AM Content (%)	MBA Content (%)	Viscosity (MPa·s)
1	2	10	0.4	3329
2	2	11	0.8	3511
3	2	12	0.6	3853
4	2.5	10	0.8	6440
5	2.5	11	0.6	6541
6	2.5	12	0.4	6673
7	3	10	0.6	9037
8	3	11	0.4	9385
9	3	12	0.8	9825

**Table 5:** Significance analysis of CS, AM, and MBA content on viscosity.

Source of Differences	Sum of Squares of Dispersion	Degree of Freedom	Mean Square	F	*p*
CS	513.646	2	256.823	106.5	0.001
AM	40.228	2	20.114	8.346	0.107
MBA	3.025	2	1.512	0.628	0.614

R^2^ = 0.999, *p* < 0.05 Significant.

**Table 6:** Material price list.

Material	Price
CS	$50–100/kg
MBA	$50–80/kg
AM	$5–15/kg
PAC	$0.5–1.5/kg
Glacial Acetic Acid	$1–2/kg
Citric Acid	$1.5–3/kg
KPS	$3–5/kg
TEMED	$100–150/kg

## Data Availability

The original contributions presented in this study are included in the article. Further inquiries can be directed to the corresponding author.
